# Peptides in Aesthetic Dermatology: Classification, Fibroblast-Centered Mechanisms, Delivery Technologies, and Clinical Evidence

**DOI:** 10.3390/life16101660

**Published:** 2026-10-02

**Authors:** Kyu-Ho Yi, Hong Seok Kim, Han Earl Lee, Won Seok Choi, Jin-Hyun Kim

**Affiliations:** 1 Department of Oral Biology, Yonsei University College of Dentistry, Seoul 03722, Republic of Korea; kyuho90@daum.net; 2 VOS Dermatology Clinic, Seoul 06021, Republic of Korea; 3 Re & Re Plastic Surgery Clinic, Seoul 06038, Republic of Korea; 4 V Plastic Surgery, Daegu 41940, Republic of Korea; 5 You and I Clinic, Seoul 06123, Republic of Korea

**Keywords:** aesthetic dermatology, bioactive peptides, collagen peptides, cosmeceuticals, dermal fibroblasts, GHK-Cu, matrikines, palmitoyl pentapeptide-4, skin aging, transdermal delivery

## Abstract

Peptides used in aesthetic dermatology differ in molecular identity, biological target, formulation, and route of administration. This critical narrative review classifies these agents and examines their direct or indirect relationships with dermal fibroblasts, integrating mechanistic, delivery, and clinical evidence. Matrikine-like and copper-binding peptides influence matrix-related responses in experimental systems, whereas neuromodulatory and pigment-modulating peptides primarily act through other cellular targets. Oral collagen hydrolysates provide amino acids and circulating hydroxyproline-containing peptides with fibroblast activity demonstrated mainly in preclinical models. Lipidation, encapsulation, and microneedles can modify exposure, but delivery-system effects complicate attribution to the peptide. Controlled human studies report improvements in selected skin endpoints, including wrinkles, hydration, elasticity, dermal density, and pigmentation; however, many trials are limited by small samples, short follow-up, industry sponsorship, or multi-ingredient formulations. Current evidence supports product-specific interpretation rather than interchangeability across peptide classes or administration routes. It does not establish universal purity thresholds, commercial shelf lives, or safety of degraded formulations. Future studies should connect characterized composition and verified exposure with cell-specific mechanisms, objective clinical outcomes, and route-appropriate safety assessment.

## 1. Introduction

Peptides are chains of amino-acid residues joined by peptide bonds. In aesthetic dermatology, this term encompasses chemically defined short sequences, lipidated derivatives, metal-binding complexes, and heterogeneous protein hydrolysates. These are not interchangeable molecular categories. KTTKS is a five-residue procollagen-derived signal, GHK is a copper-binding tripeptide, and acetyl hexapeptide-8 is modeled on part of a neuronal exocytosis protein [1,2,3]. Hydrolyzed collagen instead contains a distribution of peptide lengths and sequences; human pharmacokinetic studies identify selected hydroxyproline-containing peptides after ingestion [4,5,6]. Longer regenerative polypeptides and growth factors are considered separately because their composition and biological activity differ from those of short cosmetic peptides [7,8].

Peptides have been evaluated in topical formulations, oral supplements, dissolving microneedle patches, post-procedural regimens, and selected mesotherapy preparations [9,10,11,12,13,14]. This breadth makes the general expression “peptide technology” scientifically imprecise. A shared ingredient category does not imply a shared receptor, target tissue, delivery route, or standard of evidence. Interpretation therefore requires the exact sequence or compositional profile, chemical modification, vehicle, route, dose, and measured outcome. The clinical significance of these distinctions is particularly apparent when a peptide is studied together with other active ingredients or a device rather than against an otherwise identical peptide-free comparator [11,13,15,16].

Dermal fibroblasts provide a useful organizing center for this field. These cells synthesize and organize type I and III collagen, fibronectin, elastin-associated proteins, proteoglycans, glycosaminoglycans, and the enzymes that govern extracellular-matrix turnover. In chronologically aged skin, fibroblasts show lower matrix synthesis and weaker responsiveness to mechanical stimulation. In photoaged skin, ultraviolet radiation impairs transforming growth factor beta (TGF-β) receptor/Smad signaling, induces activator protein 1 and matrix metalloproteinases (MMPs), and fragments the collagen scaffold that normally supplies fibroblasts with tensile cues [17,18,19]. Loss of functional collagen receptors and altered endocytosis can further shift fibroblasts toward reduced type I collagen production and increased MMP-1 expression [20]. The visible endpoints relevant to aesthetics—fine wrinkling, lax texture, reduced recoil, dryness, and delayed recovery after procedures—are therefore linked to both the quantity and the organization of fibroblast-derived matrix.

A fibroblast-centered view does not imply that every aesthetic peptide acts directly on fibroblasts. Acetyl hexapeptide-8 modifies exocytosis in experimental systems, pigment-modulating peptides predominantly influence melanogenesis-related endpoints, and oral peptides undergo gastrointestinal processing before systemic exposure [3,4,5,6,21,22,23]. Reduced repetitive dermal folding is a proposed indirect consequence of neuromodulation, not proof of direct collagen induction. Similarly, improvement in pigmentation cannot establish fibroblast rejuvenation. Maintaining these distinctions prevents a clinical appearance endpoint from being assigned to an unmeasured cellular mechanism.

Peptide efficacy is also constrained by delivery. The intact stratum corneum restricts passive entry of many hydrophilic or charged molecules, and the frequently cited 500-Da rule illustrates the challenge of delivering larger compounds through skin [24]. Studies of KTTKS stability, oligoarginine conjugation, and microneedles demonstrate that molecular activity and tissue exposure are separate questions [25,26,27,28]. Formulation-level clinical improvement cannot by itself determine how much intact peptide reached living dermis or exclude contributions from the vehicle [11,15,16].

This review therefore links structural and functional classification to fibroblast biology, separates mechanistic plausibility from human efficacy, and examines delivery, quality, and safety limitations. It also considers how the recent formulation, device, and clinical studies extend the field without resolving product interchangeability or peptide-specific attribution [8,14,16,29,30,31,32,33].

This is a critical narrative review, not a systematic review or meta-analysis. A structured narrative search of PubMed/MEDLINE, Europe PMC, publisher databases, and reference lists covered database inception through 24 August 2026. Search terms combined peptides, skin aging, fibroblasts, collagen, representative sequences and classes, transdermal delivery, mesotherapy, and clinical trials. Primary mechanistic experiments, ex vivo human skin studies, human pharmacokinetic studies, and controlled trials were prioritized. Interpretation distinguishes peptide-differentiating controlled human studies, multi-ingredient formulation studies, preclinical experiments, and mechanistic inference. This descriptive hierarchy is not a validated grading instrument; heterogeneous preparations, comparators, and endpoints preclude a single pooled efficacy estimate.

## 2. Dermal Fibroblasts as the Mechanistic Hub of Aesthetic Skin Aging

### 2.1. Mechanical Context and Photoaging

Dermal fibroblasts respond to the organization and mechanical integrity of their surrounding extracellular matrix. In mechanically intact dermis, fibroblast spreading and collagen attachment support matrix synthesis. In aged skin, collagen fragmentation and reduced mechanical stimulation accompany lower procollagen production, even when fibroblasts remain viable [17,19]. Reduced endocytic collagen receptor Endo180 provides a related example of how altered matrix handling can decrease type I collagen production and increase MMP-1 expression [20]. These observations support evaluating fibroblast function together with its matrix environment rather than treating collagen production as an isolated cellular property.

Photoaging adds oxidative and signaling disturbances to this mechanical context. Ultraviolet irradiation impairs TGF-β type II receptor/Smad signaling in human skin, while fragmented collagen can promote oxidative stress and MMP-1 expression in fibroblasts [18,19]. These findings provide a rationale for examining matrix-derived signals, copper-peptide responses, and cellular stress modulation as distinct intervention points [1,2,34,35,36,37,38]. They do not establish that every peptide restores all components of the photoaging pathway.

### 2.2. Matrix Synthesis Versus Regulated Remodeling

An increase in a collagen marker is not equivalent to reconstruction of organized dermis. Experimental GHK-Cu studies report collagen synthesis, MMP-2 expression, glycosaminoglycan changes, and altered growth-factor responses, supporting a model of coordinated synthesis and turnover rather than indiscriminate inhibition of matrix degradation [1,34,35,36,37]. Palmitoyl-KTTKS has also affected wound contraction-related markers, including connective-tissue growth factor and α-smooth-muscle actin, in experimental models [39]. Accordingly, matrix quantity, organization, and contractile differentiation should be interpreted together, with the cell source and experimental context specified.

### 2.3. Limits of Experimental Generalization

The cited experiments span aged human skin, cultured human fibroblasts, mouse fibroblasts, wound models, and ex vivo tissue [17,37,38,39,40,41,42,43,44]. These systems address different questions and do not establish an equivalent response across donor ages, anatomical sites, or fibroblast populations. In particular, increased proliferation in mouse cells or expression of one matrix protein in a monolayer does not demonstrate rejuvenation of adult human facial dermis. A defensible description is therefore that a peptide is fibroblast-active under the tested conditions. Comparisons across donor ages and more tissue-representative models are research priorities, not findings established for every peptide class.

## 3. Structural and Functional Classification of Aesthetic Peptides

Table 1 organizes seven categories by representative molecular identity, dominant biological target, and relationship to fibroblasts. The classification is functional rather than mutually exclusive: GHK-Cu combines metal binding with signaling activity, lipidation modifies the delivery properties of a signal sequence, and collagen hydrolysates provide both nutritional substrate and selected circulating peptides [1,4,5,6,22,23,25,34,35,36,37,40]. Clinical efficacy outcomes are consolidated in Section 6; the following subsections address molecular identity and mechanistic evidence.

A defined lipopeptide such as palmitoyl pentapeptide-4 should not be equated with hydrolyzed collagen or a placenta-derived peptide mixture. Hydrolysates differ in peptide composition and circulating profiles, while growth-factor and placenta preparations involve distinct molecular or mixture-level exposures [5,6,8,14,22,23,25,33]. Molecular identity, modification, metal complexation, and compositional characterization are therefore necessary to interpret evidence. A claim about one preparation cannot be transferred to another solely because both are described as peptides.

### 3.1. Matrikine-like and Signal Peptides

Matrikines are biologically active fragments associated with extracellular-matrix proteins. KTTKS, the sequence Lys-Thr-Thr-Lys-Ser, is derived from the carboxyl-terminal region of the α1 chain of type I procollagen. Katayama and colleagues demonstrated promotion of extracellular-matrix production in fibroblast cultures [2]. Tendon-cell experiments subsequently linked KTTKS exposure to type I collagen and TGF-β expression [65]. The latter model supports matrix-repair signaling but is not a dermal clinical experiment.

**Figure 1:**
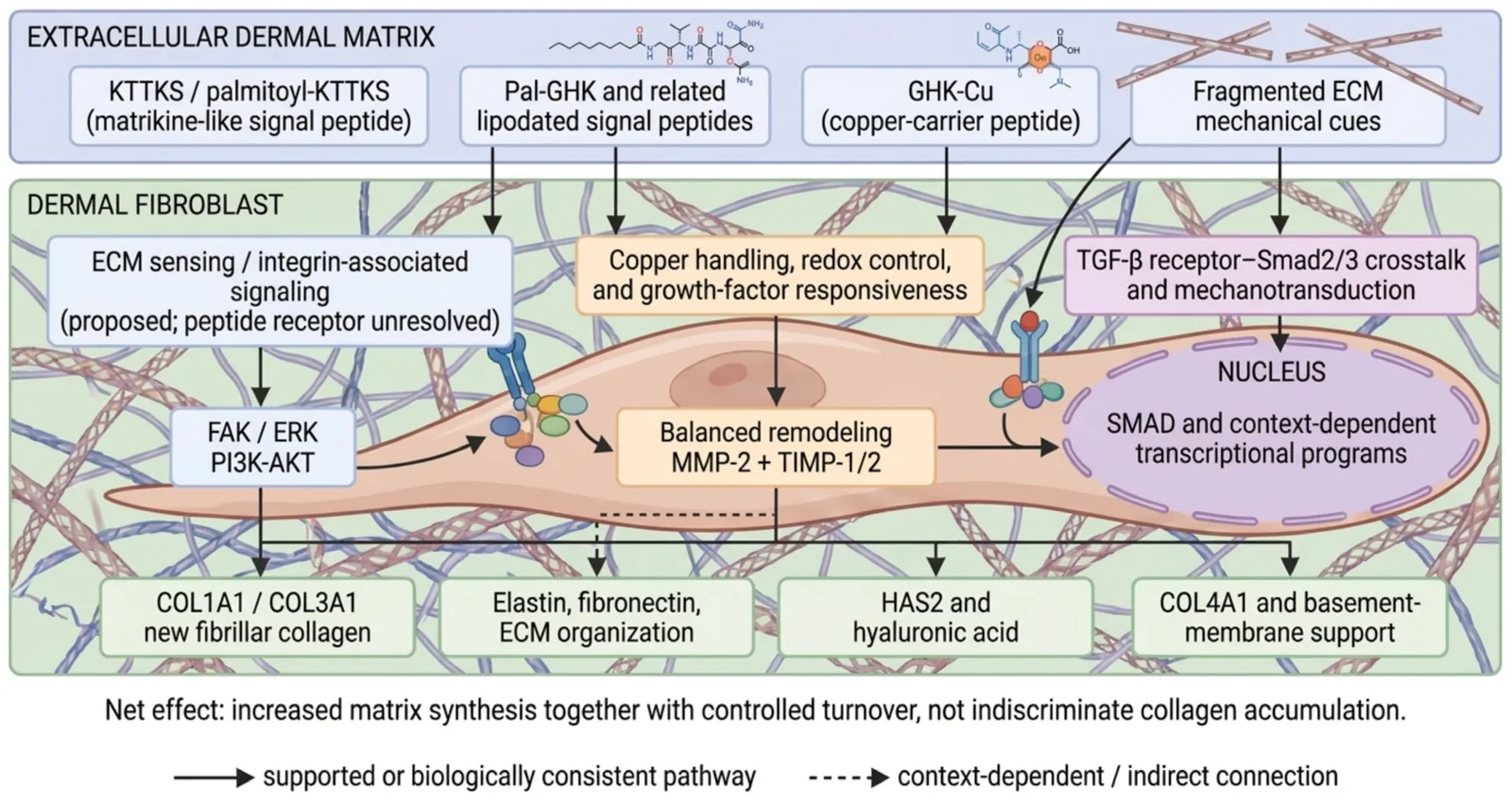
Conceptual integration of direct extracellular-matrix signaling in dermal fibroblasts. KTTKS-related signals and GHK-Cu are linked to matrix production and regulated turnover reported in experimental systems [1,2,34,35,36,37,40,65]. The cognate KTTKS receptor remains unresolved. Solid arrows indicate reported relationships or general biological compatibility, not proof that every connection has been demonstrated for each peptide in human facial skin; dashed arrows indicate proposed or context-dependent connections. HAS2—hyaluronan synthase 2; MMP—matrix metalloproteinase; TIMP—tissue inhibitor of metalloproteinases.

The cited studies support matrix-related responses without establishing a universally accepted cognate receptor for KTTKS in human dermal fibroblasts [2,40,65]. Integrin-associated mechanosensing and growth-factor crosstalk are biologically compatible with the fibroblast response, but they should not be presented as a fully verified linear peptide-specific cascade. Figure 1 therefore combines experimentally supported outputs with proposed or context-dependent upstream connections; it is a conceptual synthesis rather than a validated pathway map for a single formulation.

Palmitoylation generates the lipopeptide palmitoyl-KTTKS, commonly termed palmitoyl pentapeptide-4 and historically termed palmitoyl pentapeptide-3. A peptide-amphiphile form stimulates collagen-related responses in human fibroblasts, but dermal stability and permeation experiments show that lipidation does not eliminate degradation or distribution constraints [25,40]. Wound-contraction studies further indicate context-dependent changes in contractile differentiation [39]. Modified KTTKS sequences, including Ac-Wahx-KTTKS, represent attempts to alter sequence performance rather than evidence of class-wide equivalence [48].

Mechanistic investigations also include peptide complexes targeting basement-membrane proteins, a naturally derived peptide identified through artificial intelligence-assisted discovery, and formulations addressing cellular bioenergetics [45,46,66]. Because some of these preparations combine several ingredients, changes in cell markers should remain attached to the tested preparation. Neither a positive screening assay nor mechanistic similarity alone establishes comparable potency or clinical performance across commercial signal-peptide products.

### 3.2. Copper-Binding Peptides: GHK-Cu

GHK is the tripeptide glycyl-L-histidyl-L-lysine; its copper complex is conventionally classified as a carrier peptide. Early work established biological activity of the tripeptide, and subsequent experiments demonstrated collagen-related responses to the copper complex [1,67]. In fibroblast and wound systems, GHK-Cu has been associated with collagen synthesis, MMP-2 expression, glycosaminoglycan changes, and altered growth-factor responsiveness [1,34,35,36]. These effects are more accurately described as coordinated matrix remodeling than as a universal block of collagen degradation.

The combination of GHK-Cu and hyaluronic acid increased type IV collagen-related readouts in fibroblast and ex vivo skin tests, extending the mechanistic discussion to basement-membrane support [37]. These are not controlled facial-aging outcomes. Oligoarginine conjugation of GHK and microneedle-mediated delivery of copper peptide illustrate separate exposure-enhancing strategies [26,27]. Evidence for a GHK conjugate should not be relabeled as evidence for a copper-saturated complex unless that complex was actually studied.

GHK, GHK-Cu, and palmitoyl-GHK (palmitoyl tripeptide-1) should be named distinctly. Lipidation and copper complexation are different chemical modifications, and results from one form cannot establish the behavior of another [1,25,26]. The available biological studies also do not provide a general release specification for copper saturation, purity, or shelf stability of all marketed copper-peptide products. These quality limitations are considered in Section 7.

### 3.3. Neurotransmitter-Modulating Peptides

Acetyl hexapeptide-8, commonly known as Argireline, was designed from the amino-terminal region of synaptosomal-associated protein of 25 kDa (SNAP-25). Experimental work links it to interference with SNARE-dependent vesicle docking and neurotransmitter release [3]. This provides a mechanistic rationale for wrinkle-related investigation but does not establish penetration to the human neuromuscular target after routine topical application. The peptide should not be treated as pharmacologically equivalent to injectable botulinum toxin, and topical efficacy measurements do not prove chemodenervation [3,28,49].

The proposed fibroblast relationship is indirect. If a formulation reduced repetitive contraction, it could reduce cyclic folding and strain on the dermal matrix; that sequence remains a mechanistic inference rather than a demonstrated peptide-specific fibroblast effect in humans. Acetyl octapeptide-3 and pentapeptide-18 are discussed as related commercial concepts, not as clinically interchangeable substitutes for acetyl hexapeptide-8. The evidence summarized here does not justify assigning direct collagen stimulation or equivalent efficacy to these sequences as a group [3,28,49].

### 3.4. Proteostasis- and Redox-Modulating Peptides

Hexapeptide-11 illustrates an experimental category focused on cellular maintenance rather than imitation of a collagen fragment. In human diploid fibroblasts it modulated components of the proteostasis network and cellular responses associated with aging stress [38]. This rationale is consistent with the oxidative-stress component of fibroblast aging [19], but preservation of a cell-culture phenotype does not establish organized dermal repair in vivo. Computationally identified peptide activity similarly requires interpretation within the tested model and formulation [46].

Glutathione is a tripeptide antioxidant whose human aesthetic evidence in this review primarily concerns pigmentation or skin-condition endpoints, rather than direct proof of fibroblast collagen synthesis [50,51,52,53]. Carnosine-like peptides are often grouped with antioxidant or anti-glycation approaches, but the present reference set does not establish a comparable peptide-specific clinical matrix effect for those compounds. The terms “senomorphic” and “senolytic” should also not be used interchangeably: the cited hexapeptide-11 experiment does not demonstrate selective clearance of senescent dermal fibroblasts in humans [38].

### 3.5. Pigment-Modulating Peptides

Decapeptide-12 is a tyrosinase-inhibiting oligopeptide studied in pigment-directed formulations, while Pro-Lys-Glu-Lys (PKEK; tetrapeptide-30) has been investigated for modulation of pigmentation-related responses [21,54,55,56,57,58]. Their inclusion in an aesthetic review does not make fibroblasts their principal target. The PKEK experimental and in vivo findings establish a pigmentation context, not a surrogate measure of collagen production [21,58]. Delivery optimization of a structurally modified pigment-related peptide further illustrates the importance of the formulation and exposure conditions [59].

Pigment outcomes and matrix outcomes should therefore be evaluated separately. The studies discussed in Section 6 use melasma, dyschromia, brightness, or related skin-condition endpoints; these cannot establish increased dermal collagen or equivalence to a signal-peptide mechanism [21,50,51,52,53,54,55,56,57,58]. Where a protocol includes acids or a delivery procedure, the observed response remains a result of the combined intervention unless the comparator isolates the peptide contribution.

### 3.6. Oral Collagen-Derived Peptides

Human pharmacokinetic studies identify hydroxyproline-containing peptides in blood after gelatin or collagen hydrolysate ingestion, including Pro-Hyp and Hyp-Gly [4,5,6]. Different collagen sources generate different circulating peptide profiles [5]. Transport studies and stable-isotope methods extend the exposure evidence, but human blood detection and preclinical skin distribution are distinct observations and should not be conflated [22,23]. Oral hydrolysates therefore provide amino acid substrate together with selected circulating di- and tripeptides; this does not establish that every peptide in a supplement reaches human facial dermis intact.

**Figure 2:**
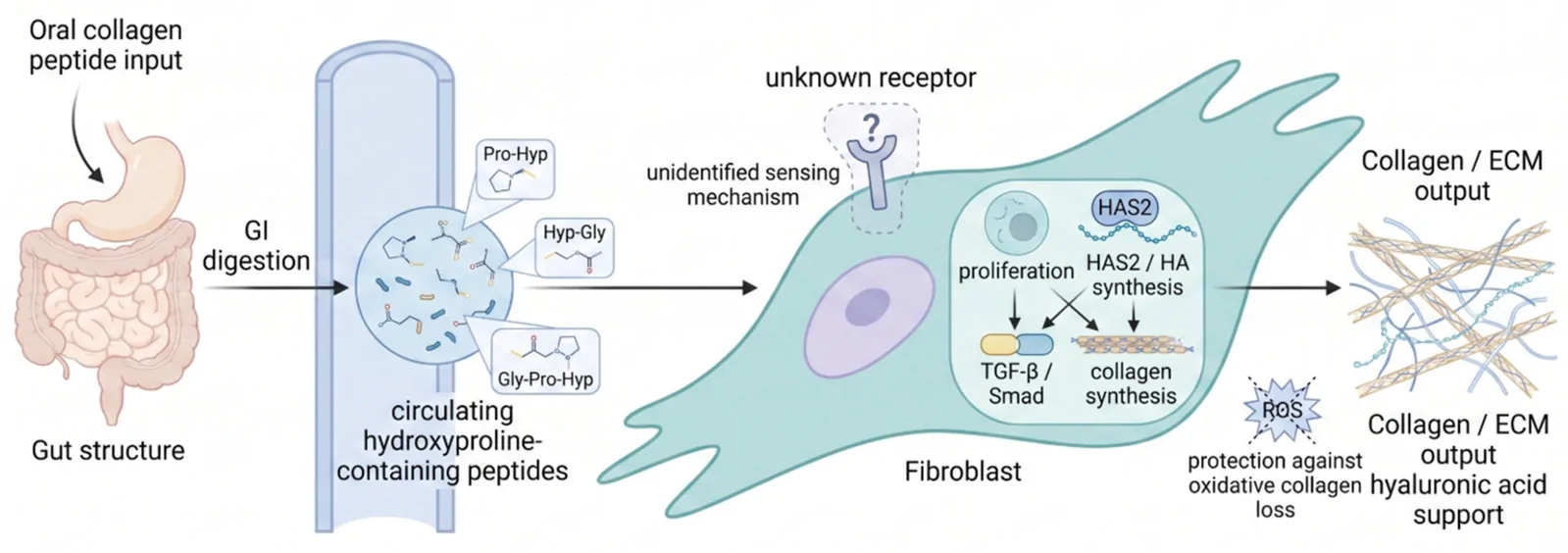
Oral collagen peptide–fibroblast axis. Human studies demonstrate selected hydroxyproline-containing peptides in blood after ingestion [4,5,6]. Skin-distribution and fibroblast-response findings include preclinical models, which should not be interpreted as a direct demonstration of the entire pathway in human facial skin [22,23,41,42,43,44,60]. Pro-Hyp, Hyp-Gly, and Gly-Pro-Hyp-related sequences are associated with proliferation, hyaluronic acid synthesis, matrix-related signaling, and oxidative-stress responses under the tested conditions. Peptide-sensing mechanisms remain incompletely defined.

Pro-Hyp enhanced growth of mouse skin fibroblasts and stimulated proliferation and hyaluronic acid synthesis in cultured human dermal fibroblasts [41,42]. Asai and colleagues studied Pro-Hyp and Hyp-Gly in primary mouse skin fibroblasts using serum free from hydroxyprolyl peptides, an important experimental qualification [43]. Other preclinical work linked collagen peptides to TGF-β/Smad-related repair and reduced collagen degradation, while a later study examined systemic Gly-Pro-Hyp absorption together with protection against hydrogen peroxide-induced fibroblast aging [44,60]. Figure 2 integrates these findings without assigning a complete receptor set or equating preclinical signaling with demonstrated human dermal remodeling.

### 3.7. Regenerative Polypeptides, Growth Factors, and Peptide Mixtures

Thymosin β4 is a 43-amino-acid actin-binding peptide investigated in tissue repair. Its wound-healing literature concerns a different biological setting from improvement of normal facial aging [7]. Epidermal growth factor (EGF), sometimes described using cosmetic oligopeptide nomenclature, is a growth factor rather than a short matrikine. The topical EGF comparison included in this review should therefore be interpreted separately from KTTKS or GHK-Cu [8]. Receptor-mediated repair concepts do not establish a shared manufacturing, stability, or safety profile across these molecules.

Mesotherapy formulations and placenta-derived preparations add another level of complexity because they contain mixtures rather than a single fully specified sequence [13,14,33]. The composition, administration route, and co-interventions determine what can be inferred. Human outcomes from these studies are presented in Section 6, while route-specific safety and quality limitations are addressed in Section 7. Evidence should remain attached to the exact formulation and route studied.

## 4. Integrated Fibroblast-Centered Mechanisms of Action

### 4.1. Direct Matrix-Related Responses

Figure 1 places matrix-synthesis and remodeling findings within the mechanical and growth-factor environment of dermal fibroblasts. The strongest peptide-specific experimental observations concern measured outputs such as collagen, basement-membrane proteins, glycosaminoglycans, or MMP-related turnover [1,2,34,35,36,37,40,45,65]. The diagram does not imply that all illustrated intermediates have been causally demonstrated for every sequence. This distinction is important when translating a convergent biological model into a claim for an individual product.

### 4.2. Oral Exposure and Experimental Fibroblast Responses

Figure 2 separates systemic exposure from cellular response. Detection of food-derived peptides in human blood establishes bioavailability of those measured species, whereas cultured-cell responses and preclinical distribution studies address subsequent steps [4,5,6,22,23,41,42,43,44,60]. A clinical hydration or elasticity benefit cannot establish which peptide or receptor caused the response. Demonstrating the complete oral peptide–human dermis pathway remains a different evidentiary task from showing a favorable endpoint in a supplement trial.

### 4.3. Indirect Mechanical and Cellular-Stress Pathways

**Figure 3:**
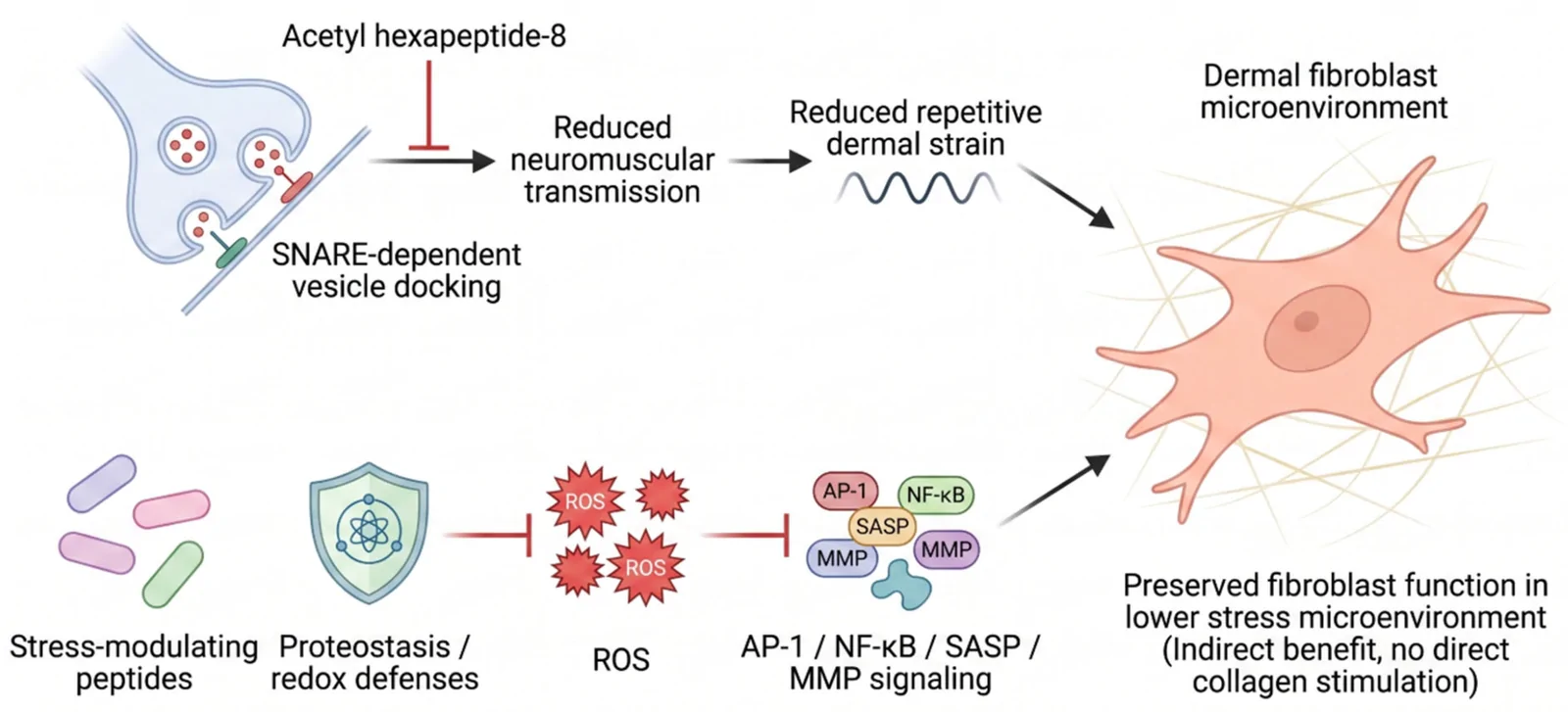
Proposed mechanical and cellular-stress relationships around dermal fibroblasts. Experimental acetyl hexapeptide-8 activity concerns exocytosis, and hexapeptide-11 findings concern fibroblast proteostasis [3,38]. The intervening connections and the final preservation of fibroblast function are conceptual interpretations, not a clinically validated causal sequence. In particular, decreased neuromuscular transmission and reduced dermal strain have not been established as the peptide-specific explanation for every topical wrinkle response. ROS—reactive oxygen species; AP-1—activator protein 1; NF-κB—nuclear factor κB; SASP—senescence-associated secretory phenotype.

Figure 3 combines the proposed mechanical consequence of neuromodulation with experimental cellular-stress modulation. SNAP-25-related exocytosis findings and hexapeptide-11 proteostasis findings arise from different experimental systems [3,38]. Any connection from reduced contraction to preserved fibroblast function remains inferential, while the stress-response branch is supported mainly by cell-level observations. Neither branch establishes a universal collagen-stimulating action for all cosmetic peptides.

A formulation may combine a signal peptide, an antioxidant peptide, a humectant, and a barrier-supporting lipid. Such combinations complicate attribution because the measured response can reflect several components rather than one molecular mechanism [15,16,66]. Endpoint selection and follow-up should therefore match the claim: surface smoothing, hydration, pigment change, and organized matrix remodeling are not interchangeable outcomes. The studies reviewed here do not establish a single time course applicable to all of them.

## 5. Delivery Technologies and Formulation Determinants

### 5.1. Passive Permeation and Lipidation

Passive topical delivery is a major translational limitation. Molecular size, polarity, charge, and the stratum corneum constrain exposure; the 500-Da rule is a useful empirical guide rather than an absolute cutoff [24]. Direct addition of a peptide to cell culture medium therefore cannot establish an equivalent concentration in living dermis after topical use. Studies of KTTKS and palmitoyl-KTTKS specifically show why both degradation and skin distribution must be assessed [25].

Lipidation changes partitioning and formulation behavior but does not guarantee delivery to the intended dermal compartment. Palmitoyl-KTTKS has been examined both as an amphiphile and within liposomes, linking molecular modification with distinct delivery systems [25,40,68]. These experiments support formulation-specific investigation; they do not establish that maximum lipophilicity, encapsulation alone, or the same nominal peptide concentration produces equivalent dermal exposure across products.

### 5.2. Molecular Modification and Vesicular Systems

Oligoarginine conjugation of GHK and molecular modification of anti-wrinkle peptides have improved delivery-related measurements in experimental studies [26,28]. Enhanced retention and permeation following structural modification, chemical enhancement, and microneedles further demonstrate the importance of delivery [59]. However, the modified molecule or combined system is not identical to the parent peptide. Comparisons should therefore retain the tested sequence, carrier, model, and endpoint rather than transferring the findings to every product carrying a related ingredient name.

The palmitoyl-KTTKS liposome study provides a recent example of physicochemical characterization linked to fibroblast responses [68]. Carrier size, loading, release, and stability characterize the investigated formulation but should not be confused with the manufacturing yield of purified peptide or a commercial expiry date. In this review, vesicular and nanoparticulate delivery is therefore treated as an exposure-modifying strategy whose clinical relevance depends on the final vehicle and comparator, not as independent proof of peptide-specific efficacy.

### 5.3. Microneedles and Procedure-Assisted Delivery

Microneedles provide a physical route through the cutaneous barrier, as illustrated by copper-peptide permeation experiments and peptide-loaded hyaluronic acid patch studies [11,27,30]. The carrier and device introduce additional variables, including dissolution, hydration, occlusion, and microinjury. Clinical outcomes are consolidated in Section 6, where these co-interventions are considered explicitly. A peptide-free matched device is an informative comparator for distinguishing payload activity from delivery-system effects.

Other combined protocols in the reviewed evidence include Dermalinfusion and laser-associated microneedling [13,57]. The resulting clinical response cannot be assigned to the peptide alone without appropriate controls. Stability is an additional limitation: evidence of biological activity before application does not demonstrate intact activity after fabrication, storage, or procedural exposure [25,68]. The term chemical or enzymatic degradation is more precise than using “denaturation” as a general synonym for the loss of activity of a short peptide.

### 5.4. Oral Exposure and Compositional Characterization

For oral preparations, source and hydrolysis-related composition influence which peptides appear in circulation [4,5,6,22,23]. A peptide fingerprint and molecular-weight distribution are therefore more informative than total protein content or the unqualified phrase “low molecular weight.” Human blood measurements support exposure to identified species, but comparisons between commercial hydrolysates require matched composition and outcome data. Clinical evidence is product-specific, as discussed in Section 6.5 [12,31,32,61,62,63,64].

## 6. Clinical Evidence: Demonstrated Outcomes and Limits of Inference

Table 2 summarizes representative human studies by intervention, design, reported outcome, and interpretive limitation. It is selective rather than exhaustive. Mechanistic experiments, human pharmacokinetic observations, and clinical appearance endpoints are kept distinct; an efficacy trial of a combination formulation establishes a formulation-level result unless its comparator isolates the peptide contribution.

### 6.1. Topical Signal-Peptide Formulations

A randomized split-face study in 93 women over 12 weeks reported improvement in photoaged facial-skin measures with topical palmitoyl pentapeptide [9]. Later work reported periorbital changes with multi-product regimens, anti-wrinkle effects of a basement-membrane-oriented peptide complex, and clinical testing of a naturally derived peptide identified through artificial intelligence [15,45,46]. Palmitoyl-RGD was also investigated in Korean women [47]. These findings support investigation of particular sequences or preparations, but the varied vehicles and combined regimens preclude a class-wide efficacy estimate.

More recent evidence includes a 2024 multi-component eye-cream study involving 37 participants over 12 weeks and a 2026 circadian-timed palmitoyl tripeptide-1 study involving 30 participants [16,29]. Both extend the clinical literature, but their results remain attached to the evaluated regimens. They do not establish that any palmitoyl peptide, concentration, or application schedule will reproduce the same outcome. In particular, a timing result from one formulation does not justify a universal morning–evening peptide protocol.

### 6.2. GHK-Cu After Laser Resurfacing

In the controlled CO2 laser-resurfacing study, 13 participants completed follow-up [10]. Subjective skin-quality satisfaction favored the GHK-Cu regimen, whereas objective assessment did not show a between-group advantage in erythema resolution, wrinkles, or overall skin quality. This neutral objective result is important: collagen-related activity in fibroblast cultures does not establish improved recovery in a specific procedural setting. The study should not be generalized either as proof of efficacy after every procedure or as evidence that all copper-peptide formulations are ineffective.

### 6.3. Neuromodulatory Peptides and Microneedle Patches

A randomized placebo-controlled study in 60 Chinese subjects reported improvement in periorbital wrinkle measurements after four weeks of topical acetyl hexapeptide-8 [49]. This supports a short-term formulation-specific endpoint, not equivalence to injectable botulinum toxin or confirmation of the proposed fibroblast-mechanical pathway. Clinical studies of peptide-loaded hyaluronic acid microneedles also reported wrinkle-related benefits, including a later dissolving dual-action patch study [11,30]. The device, hyaluronic acid, and occlusive effects limit attribution to the peptide payload alone.

### 6.4. Pigmentation and Skin-Condition Outcomes

The decapeptide-12 literature includes a split-face double-blind pilot in recalcitrant melasma, evaluations of a topical brightening system, an open-label glycolic acid combination, and a Dermalinfusion-associated report in skin of color [54,55,56,57]. These designs range from a controlled pilot to uncontrolled or combined-treatment observations and should not be assigned the same certainty. PKEK studies reported pigmentation-related benefits, including facial brightening in participants with skin types V–VI [21,58]. The outcomes concern pigment, not demonstrated fibroblast collagen synthesis.

Oral or topical glutathione studies similarly evaluated skin-lightening or skin-condition endpoints [50,51,52,53]. Positive findings in these settings do not establish a shared dermal matrix effect with matrikines or collagen hydrolysates. Across pigment-directed studies, co-ingredients, acids, and delivery procedures complicate attribution, while limited follow-up restricts conclusions about sustained response and recurrence [21,54,55,56,57,58].

### 6.5. Oral Collagen Preparations

Controlled studies of specific bioactive collagen peptides reported reduced eye-wrinkle volume, increased matrix-related markers, and improved elasticity or selected physiological measures [12,61]. Subsequent trials evaluated skin moisture and collagen-network measures, facial hydration and elasticity, and hydration, elasticity, or wrinkling after low-molecular-weight collagen intake [62,63,64]. The last of these included 64 participants [64]. These outcomes are encouraging but should remain linked to each characterized preparation and its comparator.

A 2024 trial in 85 East Asian women evaluated 5 g daily supplementation over 84 days and reported improvements in dermal density, moisture, elasticity, and wrinkle visibility [31]. Another 2024 trial assessed a hydrolyzed collagen–vitamin C supplement over 12 weeks and reported improvements in confocal collagen features and selected clinical measurements [32]. Source, peptide distribution, co-ingredients, sponsorship, and manufacturer involvement remain relevant to interpretation. The commonly studied 8–12-week intervals are intervention durations, not evidence of product shelf life, and the findings cannot be transferred to an uncharacterized collagen powder solely on the basis of protein content.

### 6.6. Growth Factors and Complex Peptide Preparations

A 2026 randomized double-blind pilot in 20 women compared synthetic and human-derived topical EGF preparations [8]. Its pilot size and growth factor-specific context limit extrapolation to short cosmetic peptides. The thymosin β4 literature includes wound-healing observations in preclinical models and patients, but those indications are not interchangeable with treatment of normal facial aging [7].

A 15-woman melasma study combined Q-switched Nd:YAG laser with microneedling and a biomimetic-peptide mesotherapy preparation, reporting improvement after five combined sessions [13]. Without separate laser-only, device-only, and peptide-only groups, the peptide contribution cannot be isolated. A 2026 multicenter trial of injected placenta polypeptides in sensitive skin and a 2025 randomized 12-week oral porcine placenta study provide additional mixture-specific evidence [14,33]. Neither establishes equivalence among all placenta-derived preparations or safety across different administration routes.

### 6.7. Recurring Design Limitations

An active vehicle and a multi-ingredient regimen can improve appearance without identifying a peptide-specific mechanism [11,15,16,32,66]. Surface measurements may change even when the dermal matrix is unchanged; without direct matrix assessment, such measurements should not be described as proof of collagen reorganization. We therefore interpret results according to the measured endpoint and comparator rather than assuming a common mechanism across hydration, wrinkle, pigment, and histological outcomes.

Short follow-up, small samples, heterogeneous concentrations, and differences in anatomical sites restrict generalization across the cited trials [8,9,10,12,16,29,31,32,49,54,61,62,63,64]. No single minimum trial duration is established for every peptide or endpoint by this review. Longer observation and assessment after discontinuation would address durability, while prespecified outcomes, matched vehicles, and independent blinded evaluation would strengthen causal interpretation. These are design priorities derived from the limitations of the included evidence, not a retrospectively applied validated risk-of-bias score.

Commercial sponsorship does not invalidate an experiment, but it increases the importance of formulation disclosure and independent replication, particularly where co-ingredients prevent peptide-only attribution [16,29,31,32]. Neutral results should be retained alongside favorable studies, as illustrated by the objective findings after CO2 laser resurfacing [10]. Overall, the evidence supports measured, preparation-specific changes in appearance rather than interchangeable or uniformly dramatic structural rejuvenation.

## 7. Safety, Quality, Manufacturing, and Stability Considerations

### 7.1. Molecular Purity and Route-Specific Suitability

Quality assessment begins with the distinction between a defined peptide and a peptide-containing mixture. Identity and chromatographic purity of a synthetic sequence are not equivalent to its concentration in a finished cream, biological potency, or microbiological suitability. For collagen hydrolysates and placenta mixtures, a single sequence-purity percentage would not adequately describe the preparation; composition and peptide distribution are central to interpretation [5,6,14,22,23,25,33,68]. Thus, comparison should specify the analytical attribute being measured rather than use “purity” as an unqualified indicator of safety.

The evidence reviewed here does not establish typically acceptable numerical purity levels that can be applied universally to oral, topical, or intradermal aesthetic peptides. The topical, oral, and invasive studies concern different preparations and exposure settings [8,9,10,12,13,14,31,32,33,61,62,63,64]. Consequently, this review does not assign route-wide percentage cutoffs or infer injection suitability from a research-grade purity certificate. A product-specific specification would need to address identity, content, impurities, stability, and microbiological quality for the intended use; these requirements cannot be replaced by a high purity percentage alone.

### 7.2. Manufacturing Approach and Production Efficiency

The preparations in this review arise from different manufacturing objectives: synthesis and modification of defined sequences, production of collagen-derived hydrolysates, and preparation of growth-factor or tissue-derived mixtures [4,5,6,8,14,22,23,33,38,48]. A method suitable for making a defined short lipopeptide is not directly comparable with one designed to obtain a broad hydrolysate distribution. Likewise, liposome preparation and peptide encapsulation are formulation operations rather than measurements of purified peptide synthesis efficiency [68].

No common head-to-head dataset on purified yield, cycle time, scale, cost, or impurity-removal burden is provided by the evidence appraised here. The evidence therefore does not identify a single most efficient manufacturing method across these molecular classes. Efficiency should be evaluated against the intended molecular or compositional specification and finished-product quality, not only crude material output. This remains a manufacturing evidence gap rather than a basis for recommending one production platform.

### 7.3. Microbial Contamination and Endotoxin

Microbiological suitability must be considered separately from molecular identity or purity. The reviewed studies distinguish intact-skin use, post-laser exposure, microneedle delivery, and injected mixtures; their safety findings cannot be transferred automatically between these routes [10,11,13,14,30]. A preparation shown to be tolerable on intact skin has not thereby been qualified for intradermal injection. Sterility, endotoxin, particulates, and container integrity are therefore critical quality questions when interpreting invasive-product evidence, rather than outcomes that can be assumed from topical efficacy.

The studies assembled here do not establish a general procedure for removing endotoxin or microbial contamination from a finished aesthetic peptide product. Their clinical outcomes must not be interpreted as validation of an improvised decontamination step. As a precautionary interpretation, a product with suspected contamination or failed quality specifications should be withheld from use pending assessment by the responsible manufacturer or quality service; the review offers no basis for treating an unvalidated filtration, heating, or preservative addition as proof that the product is safe. Numerical acceptance limits and validated corrective procedures are product- and use-specific information not established by this clinical and mechanistic evidence base.

### 7.4. Shelf Life and Storage Temperature

Experimental peptide stability and the shelf life of a marketed formulation are different concerns. KTTKS stability/permeation work and liposomal palmitoyl-KTTKS characterization demonstrate the relevance of the molecular form and delivery system [25,68], but they do not establish an expiry period for every topical, oral, or intradermal peptide product. Similarly, treatment periods reported in clinical studies are not storage studies [8,9,12,13,14,16,31,32,33,49,61,62,63,64]. A commercial shelf-life claim would require data for the final formulation and packaging, including relevant conditions after opening or reconstitution where applicable. Those product-specific datasets are not supplied by this review.

The available evidence also does not justify a universal instruction to refrigerate or freeze all peptide-containing products. Stability under a particular experimental storage condition is not proof that colder storage is superior for every sequence, emulsion, liposome, hydrolysate, or injectable mixture [25,68]. The appropriate interpretation is to use the storage conditions validated for the particular finished product, not extrapolate from an isolated peptide or another route of administration. Accordingly, no generic temperature range or shelf-life duration is assigned here.

### 7.5. Degradation Products and Potential Toxicity

Chemical or enzymatic degradation, loss of biological activity, and microbiological contamination are distinct concerns. For short peptides, hydrolysis or oxidation is more informative terminology than describing every change as “denaturation.” The reviewed stability and formulation experiments establish that peptide integrity and delivery can change, but do not provide a route-wide toxicological catalogue of aged commercial products [25,68]. Loss of activity alone neither demonstrates toxicity nor establishes that all resulting species are harmless.

Physiological generation and absorption of collagen-derived di- and tripeptides provide evidence about digestion and selected peptide responses [4,5,6,22,23,41,42,43,44,60]. They do not show that degradation products formed during storage of a topical or intradermal formulation are safe. The cited clinical trials likewise evaluate the administered preparations, not deliberately degraded versions [8,9,10,12,13,14,31,32,33,49,61,62,63,64]. Thus, the present evidence supports neither the blanket claim that degradation necessarily generates toxic skin-active molecules nor a blanket reassurance that an aged formulation remains safe. Product-specific degradation profiling and safety assessment remain necessary to resolve that question.

### 7.6. Clinical Tolerability and Scope of Safety Claims

Safety observations must remain tied to the tested formulation, route, population, and duration [8,10,11,12,13,14,16,30,31,32,33,49,50,51,52,53,61,62,63,64]. Lipidated peptides and conjugates are chemically distinct from their parent sequences; tolerability of one form cannot simply be assigned to another [25,26,28]. Multi-ingredient formulations also prevent an adverse reaction from being attributed to the peptide without further assessment. The reviewed short-term studies do not establish long-term safety across all products, repeated invasive exposure, or special populations not represented in the trials.

For oral preparations, collagen source and mixture composition are relevant to interpretation and to manufacturers’ disclosure of source allergens [5,12,31,32,33,61,62,63,64]. For growth-factor and placenta-derived preparations, the evidence does not establish regulatory interchangeability with short cosmetic peptides or a universal authorization for aesthetic injection [7,8,13,14,33]. This review therefore reports study-specific evidence without assigning jurisdiction-wide regulatory status or assuming that a cosmetic ingredient is acceptable for invasive administration.

## 8. Clinical Interpretation and Evidence Limitations

A phenotype-based organization can help readers interpret the literature without functioning as a treatment protocol. For static fine lines, topical signal-peptide studies provide formulation-specific findings; for dynamic periorbital lines, acetyl hexapeptide-8 and peptide-patch studies address different interventions [9,11,15,16,29,30,45,46,47,49]. These data do not establish equivalence to fillers, biostimulators, or injectable neuromodulators. Hydration and surface texture are also influenced by the vehicle, so a favorable appearance endpoint should not automatically be described as dermal matrix rebuilding [11,16,32].

Post-procedural claims need particular restraint. The objective GHK-Cu findings after CO2 laser resurfacing do not support a general assertion of accelerated recovery [10], and the melasma laser–microneedling study cannot isolate the biomimetic-peptide contribution [13]. Pigment-directed studies concern particular peptide systems and endpoints, not validated substitution for another treatment or proof of collagen induction [21,54,55,56,57,58]. Table 3 retains the clinically useful phenotype framework while identifying the evidence and its limits rather than prescribing product combinations or timing.

Selected oral collagen trials support modest changes in hydration, elasticity, wrinkle measures, or matrix-related endpoints, but comparisons across sources and hydrolysates remain limited [5,12,31,32,61,62,63,64]. An outcome from a characterized preparation should not be transferred to a product sharing only the collagen label. Likewise, the 2026 circadian study provides a regimen-specific observation rather than justification for a universal morning–evening schedule or post-procedural reintroduction interval [29]. Such schedules and substitutions are not established by the present evidence.

Clinical documentation should distinguish patient-reported appearance from instrumental, pigment, and matrix-related outcomes. The diversity of measures across the trials explains why one endpoint cannot substitute for another [9,10,12,16,31,32,49,54,61,62,63,64]. Standardized photography, consistent expression and lighting, and prespecified measurements are proposed here to improve interpretability, not as evidence that any particular peptide regimen has already met a uniform reporting standard.

## 9. Research Priorities and Future Directions

### 9.1. Connecting Molecular Identity, Exposure, and Cell Response

KTTKS matrix-response studies, GHK-Cu remodeling experiments, and collagen-derived peptide work identify measurable biological activity while leaving important receptor and context questions unresolved [1,2,9,34,35,36,37,40,41,42,43,44,60,65]. Future experiments should connect sequence identity and stability in biological matrices with concentration–response relationships and tissue-representative endpoints. Comparisons of young and aged human fibroblasts, three-dimensional skin systems, and cell-resolved approaches are proposed research strategies; the current evidence does not establish their results for all seven peptide categories.

Delivery studies show that modification, conjugation, encapsulation, and microneedles can change the experimental exposure problem [25,26,27,28,59,68]. Priority should be given to measuring intact peptide at the intended target and separating delivery-system effects from payload activity. Computational discovery offers a complementary approach, as illustrated by the naturally derived peptide already discussed [46], but favorable screening results still require experimental and clinical validation. Sequence discovery and delivery optimization should be evaluated as complementary, product-specific strategies.

### 9.2. Trial Design, Durability, and Independent Replication

The small or formulation-specific studies summarized in Table 2 motivate matched comparators, prespecified outcomes, independent blinded assessment, and follow-up after treatment cessation [8,9,10,16,29,31,32,49,54]. For combined devices and peptides, separate component controls would clarify attribution [11,13,30]. For oral hydrolysates, head-to-head studies should compare characterized peptide profiles rather than total grams alone, linking human exposure measurements to clinically meaningful outcomes [4,5,6,12,22,23,31,32,61,62,63,64]. These proposed design improvements address the principal limitations of the included studies.

### 9.3. Product Characterization and Reporting

The quality gaps in Section 7 require product-level evidence on impurities, microbial control, storage, and degradation rather than extrapolation from cell potency or clinical treatment duration [25,68]. Future reports should state sequence or compositional fingerprint, modification, analytical purity or content as appropriate, dose, vehicle, route, storage conditions, comparator, outcome schedule, adverse events, funding, and manufacturer role. This proposed reporting framework would make peptide-specific and formulation-specific conclusions easier to distinguish; it is not presented as an established consensus standard.

## 10. Conclusions

The principal contribution of a fibroblast-centered classification is not to assign one anti-aging mechanism to all peptides, but to distinguish direct experimental matrix responses from indirect effects and formulation-level clinical outcomes. The reviewed studies support biological activity and selected improvements with particular topical and oral preparations. They do not establish interchangeability among sequences, hydrolysates, growth factors, or administration routes. Clinical appearance alone cannot verify the proposed receptor pathway or demonstrate organized dermal remodeling.

Translation depends on connecting characterized composition with intact exposure, an appropriate comparator, a relevant endpoint, and safety under the intended conditions of use. The purity percentages, commercial shelf lives, optimum storage temperatures, manufacturing efficiency, and safety of degraded products cannot be generalized from this evidence base. The next step is therefore independent, product-specific research that integrates mechanism, durability, and quality assessment, while preserving neutral findings and distinguishing proposed explanations from demonstrated human effects. Until those links are established, measured interpretation is more defensible than either class-wide enthusiasm or dismissal.

## Figures and Tables

**Table 1:** Structural and functional classification of aesthetic peptides. References in each row support the representative examples and experimental or clinical context; the stated limitations are the interpretation applied in this review.

Class	Representative Examples/References	Primary Target	Fibroblast Relevance	Main Evidentiary Limitation
Matrikine/signal	KTTKS; palmitoyl-KTTKS; related signal peptides [2,15,25,39,40,45,46,47,48]	Matrix-related signaling and protein expression	Direct experimental collagen, fibronectin, and basement-membrane responses	Receptor and exposure uncertainties; multi-ingredient clinical testing
Copper carrier	GHK-Cu [1,10,27,34,35,36,37]	Copper-associated signaling and matrix turnover	Experimental collagen, GAG, MMP-2, and growth-factor responses	Preclinical activity does not establish facial or post-procedural benefit
Neuromodulatory	Acetyl hexapeptide-8; related commercial sequences [3,11,28,30,49]	SNARE-dependent exocytosis in experimental systems	Proposed indirect reduction of repetitive dermal strain	Human neuromuscular delivery and direct fibroblast effects remain unproven
Proteostasis/redox	Hexapeptide-11; glutathione [38,50,51,52,53]	Protein quality control and redox-related responses	Cell-level stress modulation; pigment outcomes for glutathione	Not proof of clinical collagen induction or senescent-cell clearance
Pigment-modulating	Decapeptide-12; PKEK/tetrapeptide-30 [21,54,55,56,57,58,59]	Melanogenesis-related signaling	Primarily non-fibroblast targets	Small or combined-treatment studies; pigment change is not collagen evidence
Oral collagen-derived	Pro-Hyp; Hyp-Gly; Gly-Pro-Hyp-related hydrolysates [4,5,6,12,22,23,31,32,41,42,43,44,60,61,62,63,64]	Systemic exposure to selected peptides and amino acids	Experimental proliferation, HA synthesis, and matrix signaling	Composition and exposure differ among sources and hydrolysates
Regenerative/growth factor	Thymosin β4; EGF; placenta peptide mixtures [7,8,13,14,33]	Repair or receptor-mediated growth responses	Molecule- and model-dependent direct or paracrine effects	Different indications, complex mixtures, and route-specific uncertainty

**Table 2:** Representative human clinical evidence and limits of inference. Sample sizes and treatment periods describe the cited studies, not general dosing, shelf-life, or safety specifications.

Intervention	Study Design/Population	Reported Signal	Interpretive Caution
Palmitoyl-KTTKS [9]	Randomized split-face; 93 women; 12 weeks	Improved photoaged facial-skin measures	Older formulation study; penetration and concentration remain product specific
Palmitoyl-RGD [47]	Topical clinical study in Korean women	Reduced facial wrinkle measurements	Independent replication and dose–response data are limited
Multi-peptide eye cream [16]	Prospective study; 37 participants; 12 weeks	Improved wrinkles, texture, hydration-related parameters	Multiple active ingredients prevent peptide-specific attribution
Circadian palmitoyl tripeptide-1 regimen [29]	Clinical study; 30 participants	Improved collagen-related and clinical endpoints with the tested timed regimen	Regimen-specific result; does not isolate a universal timing or peptide effect
GHK-Cu after CO2 laser [10]	Controlled post-resurfacing study; 13 completed participants	Subjective satisfaction favored GHK-Cu; no objective between-group advantage	Neutral objective erythema, wrinkle, and skin-quality findings; context specific
Acetyl hexapeptide-8 [49]	Randomized placebo-controlled; 60 Chinese subjects; 4 weeks	Reduced periorbital wrinkle measurements	Short duration; formulation and blinding questions warrant replication
Peptide microneedle patch [11,30]	Controlled clinical studies; one later trial included 24 subjects	Improved wrinkle-related measures with dissolving HA microneedles	Microneedle injury, HA, and occlusion are active co-interventions
PKEK [21,58]	In vitro plus facial studies including skin types V-VI	Improved pigmentation/brightness endpoints	Primary action is melanogenesis, not fibroblast collagen synthesis
Decapeptide-12 [54,55,56,57]	Controlled pilot plus small open-label/combined-treatment reports	Improved dyschromia and post-inflammatory hyperpigmentation	Different designs and certainty; acids or Dermalinfusion can confound attribution
Specific collagen peptides [12,61]	Double-blind placebo-controlled oral trials	Reduced wrinkles; improved elasticity and matrix markers	Commercially characterized peptides; not generalizable to every collagen powder
Low-molecular-weight collagen [64]	Randomized double-blind placebo-controlled; 64 participants	Improved hydration, elasticity, and wrinkling	Product source and peptide distribution determine transferability
Collagen peptides in East Asian women [31]	Randomized double-blind placebo-controlled; 85 women; 84 days	Improved dermal density, moisture, elasticity, and wrinkle visibility	Industry involvement and standardized co-skincare should be considered
Hydrolyzed collagen + vitamin C [32]	Randomized double-blind placebo-controlled; 12 weeks	Improved confocal collagen, hydration, elasticity, and wrinkles	Combination product and manufacturer-employed authors limit peptide-only attribution
Topical EGF [8]	Randomized double-blind pilot; 20 women	Facial rejuvenation and psychosocial outcomes	EGF is a growth factor, not a short cosmetic peptide; pilot scale
Biomimetic-peptide melasma protocol [13]	15 women; laser + microneedling + peptide mesotherapy	Reduced mMASI after five combined sessions	No laser-only, microneedle-only, or peptide-only control

**Table 3:** Phenotype-based interpretation of the cited evidence, not a treatment guideline. References identify the evaluated interventions; the final column states limits of inference rather than contraindication or dosing rules.

Clinical Phenotype	Evaluated Approach/References	Reported Endpoint	Limit of Inference
Fine static lines/photoaging	Topical signal-peptide formulations [9,15,16,29,45,46,47]	Wrinkle or photoaging-related measurements	Formulation-specific; no equivalence to fillers, biostimulators, or resurfacing
Dynamic periorbital lines	Acetyl hexapeptide-8; peptide-loaded HA patches [11,30,49]	Periorbital wrinkle measurements	Does not establish chemodenervation or the proposed fibroblast-mechanical pathway
Dryness/skin texture	Multi-component topicals; selected oral collagen preparations [16,31,32,62,63,64]	Hydration, elasticity, or texture measurements	Smoothing may be vehicle-driven; hydration is not proof of matrix rebuilding
Dyschromia/melasma	Decapeptide-12 or PKEK formulations [21,54,55,56,57,58]	Pigment or brightness measurements	Small or combined-treatment studies; durability and recurrence remain uncertain
Post-procedural context	GHK-Cu after CO2 laser; multimodal peptide–microneedling protocol [10,13]	Recovery-related or melasma measurements	Neutral objective GHK-Cu findings; combined protocols do not isolate peptide efficacy
Oral supplementation	Characterized collagen hydrolysates [5,12,31,32,61,62,63,64]	Hydration, elasticity, wrinkles, or matrix-related measures	No interchangeability by protein content alone; treatment duration is not shelf life

## Data Availability

No new datasets were generated. All sources are cited in the manuscript.

## References

[1] Maquart F.X., Pickart L., Laurent M., Gillery P., Monboisse J.C., Borel J.P. (1988). Stimulation of collagen synthesis in fibroblast cultures by the tripeptide-copper complex glycyl-L-histidyl-L-lysine-Cu^2+^. FEBS Lett..

[2] Katayama K., Armendariz-Borunda J., Raghow R., Kang A.H., Seyer J.M. (1993). A pentapeptide from type I procollagen promotes extracellular matrix production. J. Biol. Chem..

[3] Blanes-Mira C., Clemente J., Jodas G., Gil A., Fernández-Ballester G., Ponsati B., Gutierrez L., Pérez-Payá E., Ferrer-Montiel A. (2002). A synthetic hexapeptide (Argireline) with antiwrinkle activity. Int. J. Cosmet. Sci..

[4] Iwai K., Hasegawa T., Taguchi Y., Morimatsu F., Sato K., Nakamura Y., Higashi A., Kido Y., Nakabo Y., Ohtsuki K. (2005). Identification of food-derived collagen peptides in human blood after oral ingestion of gelatin hydrolysates. J. Agric. Food Chem..

[5] Ohara H., Matsumoto H., Ito K., Iwai K., Sato K. (2007). Comparison of quantity and structures of hydroxyproline-containing peptides in human blood after oral ingestion of gelatin hydrolysates from different sources. J. Agric. Food Chem..

[6] Shigemura Y., Akaba S., Kawashima E., Park E.Y., Nakamura Y., Sato K. (2011). Identification of a novel food-derived collagen peptide, hydroxyprolyl-glycine, in human peripheral blood by pre-column derivatisation with phenyl isothiocyanate. Food Chem..

[7] Treadwell T., Kleinman H.K., Crockford D., Hardy M.A., Guarnera G.T., Goldstein A.L. (2012). The regenerative peptide thymosin beta-4 accelerates the rate of dermal healing in preclinical animal models and in patients. Ann. N. Y. Acad. Sci..

[8] Abud B., McGinn L., Cawthon E., Pankey A., Gandhi N., Carvajal C., Dayan S. (2026). A randomized, double-blinded pilot study comparing synthetic versus human-derived topical epidermal growth factor for facial rejuvenation and psychosocial perception. J. Cosmet. Dermatol..

[9] Robinson L.R., Fitzgerald N.C., Doughty D.G., Dawes N.C., Berge C.A., Bissett D.L. (2005). Topical palmitoyl pentapeptide provides improvement in photoaged human facial skin. Int. J. Cosmet. Sci..

[10] Miller T.R., Wagner J.D., Baack B.R., Eisbach K.J. (2006). Effects of topical copper tripeptide complex on CO_2_ laser-resurfaced skin. Arch. Facial Plast. Surg..

[11] Avcil M., Akman G., Klokkers J., Jeong D., Çelik A. (2020). Efficacy of bioactive peptides loaded on hyaluronic acid microneedle patches: A monocentric clinical study. J. Cosmet. Dermatol..

[12] Proksch E., Schunck M., Zague V., Segger D., Degwert J., Oesser S. (2014). Oral intake of specific bioactive collagen peptides reduces skin wrinkles and increases dermal matrix synthesis. Skin. Pharmacol. Physiol..

[13] Karadağ Köse Ö., Borlu M. (2021). Efficacy of the combination of Q-switched Nd:YAG laser and microneedling for melasma. J. Cosmet. Dermatol..

[14] Shu C., Zhang X.Y., Han X.F., Liu J.-W., Sun X., Sun Q.-N. (2026). Safety and efficacy of mesotherapy with placenta polypeptide injection for moderate-to-severe sensitive skin: A multicenter, randomized, controlled trial. Clin. Cosmet. Investig. Dermatol..

[15] Kaczvinsky J.R., Griffiths C.E.M., Schnicker M.S., Li J. (2009). Efficacy of anti-aging products for periorbital wrinkles as measured by 3-D imaging. J. Cosmet. Dermatol..

[16] Yang F., Zhang X., Wang H., Guo M., Zhang J., Feng X., Yu J., Yang J., Zhu J., Wang Y. (2024). Comprehensive evaluation of the efficacy and safety of a new multi-component anti-aging topical eye cream. Skin. Res. Technol..

[17] Varani J., Dame M.K., Rittie L., Fligiel S.E., Kang S., Fisher G.J., Voorhees J.J. (2006). Decreased collagen production in chronologically aged skin: Roles of age-dependent alteration in fibroblast function and defective mechanical stimulation. Am. J. Pathol..

[18] Quan T., He T., Kang S., Voorhees J.J., Fisher G.J. (2004). Solar ultraviolet irradiation reduces collagen in photoaged human skin by blocking transforming growth factor-beta type II receptor/Smad signaling. Am. J. Pathol..

[19] Fisher G.J., Quan T., Purohit T., Shao Y., Cho M.K., He T., Varani J., Kang S., Voorhees J.J. (2009). Collagen fragmentation promotes oxidative stress and elevates matrix metalloproteinase-1 in fibroblasts in aged human skin. Am. J. Pathol..

[20] Iwahashi H., Kawashima Y., Masaki H. (2022). Decreased levels of endocytic collagen receptor Endo180 in dermal fibroblasts lead to decreased production of type I collagen and increased expression of matrix metalloproteinase-1. Photodermatol. Photoimmunol. Photomed..

[21] Marini A., Farwick M., Grether-Beck S., Brenden H., Felsner I., Jaenicke T., Weber M., Schild J., Maczkiewitz U., Köhler T. (2012). Modulation of skin pigmentation by the tetrapeptide PKEK: In vitro and in vivo evidence for skin whitening effects. Exp. Dermatol..

[22] Yazaki M., Ito Y., Yamada M., Goulas S., Teramoto S., Nakaya M.-A., Ohno S., Yamaguchi K. (2017). Oral ingestion of collagen hydrolysate leads to the transportation of highly concentrated Gly-Pro-Hyp and its hydrolyzed form of Pro-Hyp into the bloodstream and skin. J. Agric. Food Chem..

[23] Taga Y., Iwasaki Y., Shigemura Y., Mizuno K. (2019). Improved in vivo tracking of orally administered collagen hydrolysate using stable isotope labeling and LC-MS techniques. J. Agric. Food Chem..

[24] Bos J.D., Meinardi M.M.H.M. (2000). The 500 Dalton rule for the skin penetration of chemical compounds and drugs. Exp. Dermatol..

[25] Choi Y.L., Park E.J., Kim E., Na D.H., Shin Y.H. (2014). Dermal stability and in vitro skin permeation of collagen pentapeptides (KTTKS and palmitoyl-KTTKS). Biomol. Ther..

[26] Hur G.H., Han S.C., Ryu A.R., Eom Y., Kim J.W., Lee M.Y. (2020). Effect of oligoarginine conjugation on the antiwrinkle activity and transdermal delivery of GHK peptide. J. Pept. Sci..

[27] Li H., Low Y.S.J., Chong H.P., Zin M.T., Lee C.-Y., Li B., Leolukman M., Kang L. (2015). Microneedle-mediated delivery of copper peptide through skin. Pharm. Res..

[28] Lim S.H., Sun Y., Thiruvallur Madanagopal T., Rosa V., Kang L. (2018). Enhanced skin permeation of anti-wrinkle peptides via molecular modification. Sci. Rep..

[29] Wang C., Song T., Zhang Y., Li N., Xie L., Xie M., Jiang X., Lü G., Meng Y., Wang C. (2026). Targeting circadian rhythm for the regulation of skin collagen metabolism. J. Cosmet. Dermatol..

[30] Shin J.Y., Han D.H., Yoon K.Y., Jeong D.H., Park Y.I. (2024). Clinical safety and efficacy evaluation of a dissolving microneedle patch having dual anti-wrinkle effects with safe and long-term activities. Ann. Dermatol..

[31] Vleminckx S., Virgilio N., Asserin J., Prawitt J., Silva C.I.F. (2024). Influence of collagen peptide supplementation on visible signs of skin and nail health and aging in an East Asian population: A double-blind, randomized, placebo-controlled trial. J. Cosmet. Dermatol..

[32] Reilly D.M., Kynaston L., Naseem S., Proudman E., Laceby D. (2024). A clinical trial shows improvement in skin collagen, hydration, elasticity, wrinkles, scalp, and hair condition following 12-week oral intake of a supplement containing hydrolysed collagen. Dermatol. Res. Pract..

[33] Nguyen N.H., Lee Y.I., Chau N.H., Lee S.J., Kim I., Kim J., Baek K.-S., Lee J.H. (2025). Porcine placenta peptides as a complementary functional food for skin rejuvenation: A 12-week randomized, double-blind, placebo-controlled trial. Complement. Ther. Med..

[34] Siméon A., Emonard H., Hornebeck W., Maquart F.X. (2000). The tripeptide-copper complex glycyl-L-histidyl-L-lysine-Cu^2+^ stimulates matrix metalloproteinase-2 expression by fibroblast cultures. Life Sci..

[35] Simeon A., Wegrowski Y., Bontemps Y., Maquart F.X. (2000). Expression of glycosaminoglycans and small proteoglycans in wounds: Modulation by the tripeptide-copper complex glycyl-L-histidyl-L-lysine-Cu^2+^. J. Investig. Dermatol..

[36] Pollard J.D., Quan S., Kang T., Koch R.J. (2005). Effects of copper tripeptide on the growth and expression of growth factors by normal and irradiated fibroblasts. Arch. Facial Plast. Surg..

[37] Jiang F., Wu Y., Liu Z., Hong M., Huang Y. (2023). Synergy of GHK-Cu and hyaluronic acid on collagen IV upregulation via fibroblast and ex vivo skin tests. J. Cosmet. Dermatol..

[38] Sklirou A.D., Ralli M., Dominguez M., Papassideri I., Skaltsounis A.-L., Trougakos I.P. (2015). Hexapeptide-11 is a novel modulator of the proteostasis network in human diploid fibroblasts. Redox Biol..

[39] Park H., An E., Cho Lee A.R. (2017). Effect of palmitoyl-pentapeptide (Pal-KTTKS) on wound contractile process in relation with connective tissue growth factor and alpha-smooth muscle actin expression. Tissue Eng. Regen. Med..

[40] Jones R.R., Castelletto V., Connon C.J., Hamley I.W. (2013). Collagen stimulating effect of peptide amphiphile C16-KTTKS on human fibroblasts. Mol. Pharm..

[41] Shigemura Y., Iwai K., Morimatsu F., Iwamoto T., Mori T., Oda C., Taira T., Park E.Y., Nakamura Y., Sato K. (2009). Effect of prolyl-hydroxyproline (Pro-Hyp), a food-derived collagen peptide in human blood, on growth of fibroblasts from mouse skin. J. Agric. Food Chem..

[42] Ohara H., Ichikawa S., Matsumoto H., Akiyama M., Fujimoto N., Kobayashi T., Tajima S. (2010). Collagen-derived dipeptide, proline-hydroxyproline, stimulates cell proliferation and hyaluronic acid synthesis in cultured human dermal fibroblasts. J. Dermatol..

[43] Asai T.T., Oikawa F., Yoshikawa K., Inoue N., Sato K. (2020). Food-Derived Collagen Peptides, Prolyl-Hydroxyproline (Pro-Hyp), and Hydroxyprolyl-Glycine (Hyp-Gly) Enhance Growth of Primary Cultured Mouse Skin Fibroblast Using Fetal Bovine Serum Free from Hydroxyprolyl Peptide. Int. J. Mol. Sci..

[44] Liu Z., Li Y., Song H., He J., Li G., Zheng Y., Li B. (2019). Collagen peptides promote photoaging skin cell repair by activating the TGF-beta/Smad pathway and depressing collagen degradation. Food Funct..

[45] Jeong S., Yoon S., Kim S., Jung J., Kor M., Shin K., Lim C., Han H.S., Lee H., Park K.-Y. (2020). Anti-wrinkle benefits of peptides complex stimulating skin basement membrane proteins expression. Int. J. Mol. Sci..

[46] Kennedy K., Cal R., Casey R., Lopez C., Adelfio A., Molloy B., Wall A.M., Holton T.A., Khaldi N. (2020). The anti-ageing effects of a natural peptide discovered by artificial intelligence. Int. J. Cosmet. Sci..

[47] Bae J.S., Kim J.M., Kim J.Y., Choi C.H., Kim J.Y., Moon W.K., Lee M.S., Moon S.H., Lim J.H., Park S.J. (2017). Topical application of palmitoyl-RGD reduces human facial wrinkle formation in Korean women. Arch. Dermatol. Res..

[48] Guglielmi D.A., Martinelli A.M., Rissi N.C., Cilli E.M., Soares C.P., Chiavacci L.A. (2016). Synthesis of the peptide Ac-Wahx-KTTKS and evaluation of the ability to induce in vitro collagen synthesis. Protein Pept. Lett..

[49] Wang Y., Wang M., Xiao S., Pan P., Li P., Huo J. (2013). The anti-wrinkle efficacy of argireline, a synthetic hexapeptide, in Chinese subjects: A randomized, placebo-controlled study. Am. J. Clin. Dermatol..

[50] Arjinpathana N., Asawanonda P. (2012). Glutathione as an oral whitening agent: A randomized, double-blind, placebo-controlled study. J. Dermatol. Treat..

[51] Watanabe F., Hashizume E., Chan G.P., Kamimura A. (2014). Skin-whitening and skin-condition-improving effects of topical oxidized glutathione: A double-blind and placebo-controlled clinical trial in healthy women. Clin. Cosmet. Investig. Dermatol..

[52] Weschawalit S., Thongthip S., Phutrakool P., Asawanonda P. (2017). Glutathione and its antiaging and antimelanogenic effects. Clin. Cosmet. Investig. Dermatol..

[53] Wahab S., Anwar A.I., Zainuddin A.N., Hutabarat E.N., Anwar A.A., Kurniadi I. (2021). Combination of topical and oral glutathione as a skin-whitening agent: A double-blind randomized controlled clinical trial. Int. J. Dermatol..

[54] Hantash B.M., Jimenez F. (2009). A split-face, double-blind, randomized and placebo-controlled pilot evaluation of a novel oligopeptide for the treatment of recalcitrant melasma. J. Drugs Dermatol..

[55] Hantash B.M., Jimenez F. (2012). Treatment of mild to moderate facial melasma with the Lumixyl topical brightening system. J. Drugs Dermatol..

[56] Ramírez S.P., Carvajal A.C., Salazar J.C., Arroyave G., Flórez A.M., Echeverry H.F. (2013). Open-label evaluation of a novel skin brightening system containing 0.01% decapeptide-12 in combination with 20% buffered glycolic acid for the treatment of mild to moderate facial melasma. J. Drugs Dermatol..

[57] Bhatia A., Hsu J.T.S., Hantash B.M. (2014). Combined topical delivery and Dermalinfusion of decapeptide-12 accelerates resolution of post-inflammatory hyperpigmentation in skin of color. J. Drugs Dermatol..

[58] Farwick M., Maczkiewitz U., Lersch P., Summers B., Rawlings A.V. (2011). Facial skin-lightening benefits of the tetrapeptide Pro-Lys-Glu-Lys on subjects with skin types V-VI living in South Africa. J. Cosmet. Dermatol..

[59] Chen J., Bian J., Hantash B.M., Albakr L., Hibbs D.E., Xiang X., Xie P., Wu C., Kang L. (2021). Enhanced skin retention and permeation of a novel peptide via structural modification, chemical enhancement, and microneedles. Int. J. Pharm..

[60] Chae M., Moon C.Y., Lim S.H., Yamashita Y., Yamada H., Ide M., Park C.W., Roh J., Kim W. (2023). Oral ingestion of AP collagen peptide leads to systemic absorption of Gly-Pro-Hyp, alleviating H_2_O_2_-induced dermal fibroblast aging. J. Med. Food.

[61] Proksch E., Segger D., Degwert J., Schunck M., Zague V., Oesser S. (2014). Oral supplementation of specific collagen peptides has beneficial effects on human skin physiology: A double-blind, placebo-controlled study. Skin Pharmacol. Physiol..

[62] Asserin J., Lati E., Shioya T., Prawitt J. (2015). The effect of oral collagen peptide supplementation on skin moisture and the dermal collagen network: Evidence from an ex vivo model and randomized, placebo-controlled clinical trials. J. Cosmet. Dermatol..

[63] Inoue N., Sugihara F., Wang X. (2016). Ingestion of bioactive collagen hydrolysates enhance facial skin moisture and elasticity and reduce facial ageing signs in a randomised double-blind placebo-controlled clinical study. J. Sci. Food Agric..

[64] Kim D.U., Chung H.C., Choi J., Sakai Y., Lee B.Y. (2018). Oral intake of low-molecular-weight collagen peptide improves hydration, elasticity, and wrinkling in human skin: A randomized, double-blind, placebo-controlled study. Nutrients.

[65] Tsai W.C., Hsu C.C., Chung C.Y., Lin M.S., Li S.L., Pang J.H.S. (2007). The pentapeptide KTTKS promoting the expressions of type I collagen and transforming growth factor-beta of tendon cells. J. Orthop. Res..

[66] Osborne R., Carver R.S., Mullins L.A., Finlay D.R. (2013). Practical application of cellular bioenergetics to the care of aged skin. Br. J. Dermatol..

[67] Pickart L., Thayer L., Thaler M.M. (1973). A synthetic tripeptide which increases survival of normal liver cells, and stimulates growth in hepatoma cells. Biochem. Biophys. Res. Commun..

[68] Vitali A., Paolicelli P., Bigi B., Trilli J., Di Muzio L., Carriero V.C., Casadei M.A., Petralito S. (2024). Liposome Encapsulation of the Palmitoyl–KTTKS Peptide: Structural and Functional Characterization. Pharmaceutics.

